# Quantitative study of bioinformatics analysis on glioma: a bibliometric analysis

**DOI:** 10.3389/fonc.2023.1222797

**Published:** 2023-11-15

**Authors:** Xiaobing Yang, Dulegeqi Man, Peng Zhao, Xingang Li

**Affiliations:** ^1^ Department of Neurosurgery, Qilu Hospital, Cheeloo College of Medicine and Institute of Brain and Brain-Inspired Science, Shandong University, Jinan, China; ^2^ Jinan Microecological Biomedicine Shandong Laboratory and Shandong Key Laboratory of Brain Function Remodeling, Jinan, China; ^3^ Shandong Key Laboratory of Brain Function Remodeling, Jinan, China; ^4^ Department of Neurosurgery, International Mongolia Hospital of Inner Mongolia, Hohhot, China

**Keywords:** bibliometric analysis, glioma, bioinformatic, VOSviewer, Citespace

## Abstract

**Background:**

The bioinformatics analysis on glioma has been a hot point recently. The purpose of this study was to provide an overview of the research in this field using a bibliometric method.

**Methods:**

The Web of Science Core Collection (WOSCC) database was used to search for literature related to the bioinformatics analysis of gliomas. Countries, institutions, authors, references, and keywords were analyzed using VOSviewer, CiteSpace, and Microsoft Excel software.

**Result:**

China was the most productive country, while the USA was the most cited. Capital Medical University had the largest number of publications and citations. Institutions tend to collaborate more with other institutions in their countries rather than foreign ones. The most productive and most cited author was Jiang Tao. Two citation paths were identified, with literature in basic research journals often cited in clinical journals. Immune-related vocabularies appeared frequently in recent studies.

**Conclusion:**

Glioma bioinformatics analyses spanned a wide range of fields. The international communication in this field urgently needs to be strengthened. Glioma bioinformatics approaches are developing from basic research to clinical applications. Recently, immune-related research has become a focus.

## Introduction

Glioma is the most common malignant tumour of the central nervous system, accounting for 81% of all malignant tumours. Among them, glioblastoma, which has the highest degree of malignancy, has a 5-year survival rate of only 5% (1). Currently, the main treatment methods for glioma are surgery, radiation therapy, and systemic drug therapy (2); However, these existing methods cannot cure gliomas completely (2).

Bioinformation emerged 50 years ago, and DNA analysis emerged with the development of molecular biology and computer science methods (3). In recent years, the observation of intratumoral heterogeneity has led to the realization there is a need to move towards precision medicine to improve patient prognosis (4). Omics analyses are considered the key to promoting the development of precision medicine (5). In recent years, scholars have used bioinformatics methods to analyse omics data and search for glioma-related pathways and prognostic markers (6–9); predict the influence of biological process-related markers, such as ferroptosis- and pyroptosis-related markers on the prognosis of glioma (10–13); predict the mechanism of action of individual genes (14–16), including noncoding RNA (17, 18); guide the design of glioma-related experiments (19–21); search for prognostic markers in cerebrospinal fluid circulation to guide personalized treatment for clinical patients (22–24); predict the mechanism of anticancer drugs (25–28), and search for potential antiglioma drugs (16, 29–31).

Bibliometrics uses mathematical and statistical methods to quantitatively analyse knowledge carriers (32) and is a combination of science and art (33). Scholars use bibliometrics methods to analyse the number of studies published in various fields, collaborations and citation relationships between authors, countries, and institutions, and the cooccurrence of keywords. These analysis results provide insight into the development context of the studied discipline, the research foci in the field, and potential research partners (34–37).

Here, the bibliometric method was used to analyse the literature related to bioinformatics analyses of glioma to find the key content, the development frontier of this field, and the status of communication and cooperation between scholars in this field.

## Methods

### Data source and collection

We searched for relevant literature on bioinformatics analyses of glioma in the Web of Science Core Collection on April 24, 2023. The search terms we used are as follows:


(1) $$\begin{array}{c} \left(\left(\left(\text{TS}=\left(\text{glioma}\right)\right)\text{ OR TS}=\left(\text{glioblastoma}\right)\right)\text{ OR TS}=\left(\text{GBM}\right)\right)\text{ AND }\\ \left(\left(\left(\left(\text{TS}=\left(\text{bioinformatics}\right)\right)\text{ OR TS}=\left(\text{TCGA}\right)\right)\text{ OR TS}=\left(\text{CGGA}\right)\right)\text{ OR TS}=\left(\text{GEO}\right)\right) \end{array}$$

**Figure 1:**
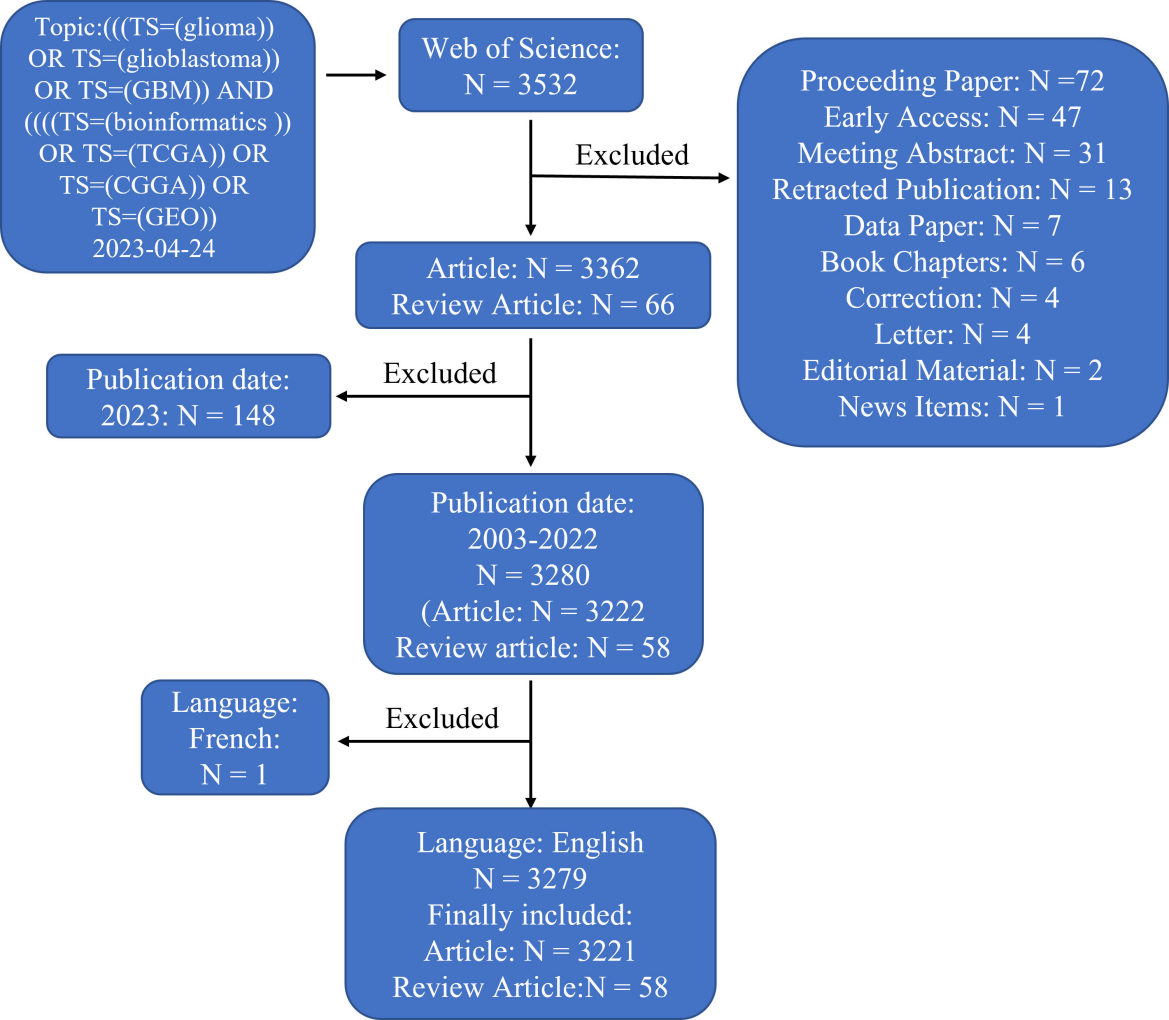
Flowchart of the analysed and excluded articles.

A total of 3532 articles were acquired. We limited the literature type to articles or review articles published in English from 2003-2022, yielding 3279 articles (3221 articles and 58 review articles; **Figure 1**). Two authors independently completed all literature searches, and the results were compared. Any differences were discussed with a third author to determine which articles would be included in the final analysis.

### Data processing and visual analysis

Bar charts and line charts were generated using Microsoft Office Excel software (Version 2021, Microsoft Corporation, Redmond, WAS, USA).

VOSviewer is a freely available computer software that visualizes bibliometric maps (38) and can be used for clustering analyses of publications, keywords, authors, institutions, and references (39). We used VOSviewer software (Version 1.6.19, Center for Science and Technology Studies, University of Leiden) to analyse and visualize coauthorships, citation relationships and cocitation relationships across countries, organizations, authors, references and keywords. The operation process is as follows. Export the literature from the WOSCC database and import it into the VOSviewer software. Select country, institution, or author from “the Unit of analysis”. Then, choose Co-authorship or Co-occurrence or Citation from “Type of analysis”. Next, set restrictions on the minimum number of publications for authors, institutions, and countries, the minimum number of citations for references, and the minimum number of occurrences for keywords. Finally, set resolutions and the minimum size of clusters. After the software automatically analyzes and visualizes the results, export the images and relevant data.

We used CiteSpace 6.2. R2, a powerful bibliometric analysis tool (40) that can run burst detection and overlay functions, to visualize the top 25 references and generate an overlay map. The operation process is as follows.After importing the literature into CiteSpace software, select “Reference” and choose “Pathfinder” and “Pruning sliced networks” for analysis. Click on “Citation/Frequency Burst” to obtain the top 25 references with the strongest citation burst. Then, we select “Overlay Maps” from the CiteSpace software, click on “Overlay”, import data, select “Z Scores”, and export the image.

## Result

### Annual growth trend of publications

**Figure 2:**
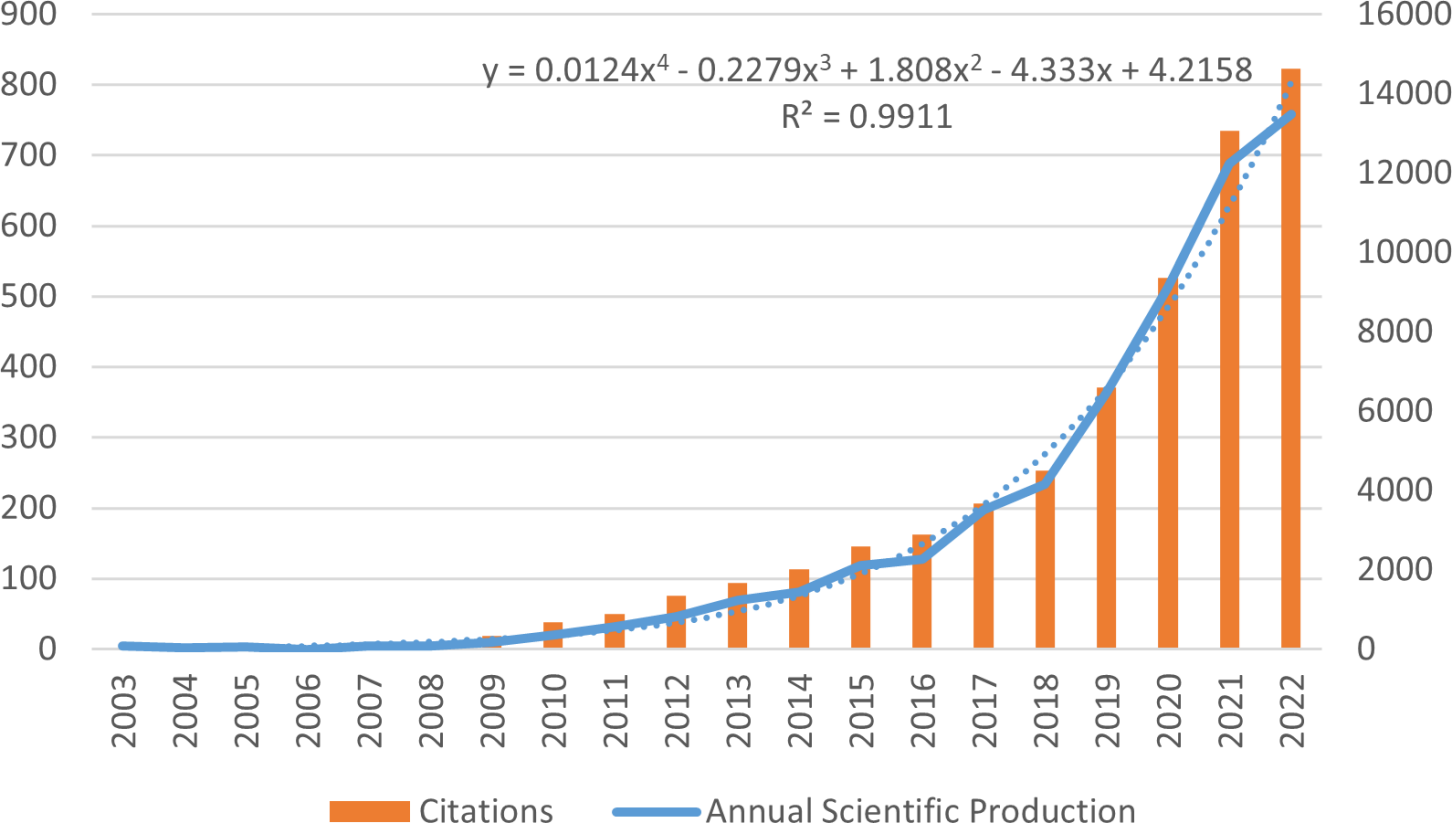
Trends in publications and citations.

We ultimately obtained 3279 eligible articles from the Web of Science database. On the date of accession (April 24, 2023), the total citation number was 67,500, with an average citation frequency of 20.59 per article and an average H-index of 94. As shown in **Figure 2**, we found that bioinformatics analyses of gliomas have steadily increased in number since 2003, and this growth has accelerated significantly since 2017, indicating that this area of research is becoming a focus. Moreover, citations of these articles increased annually (**Figure 2**), indicating that this research topic has increasingly received widespread attention and has become a focus of communication within and outside the field.

### Analysis of the distribution and cooperation of leading countries/regions

**Figure 3:**
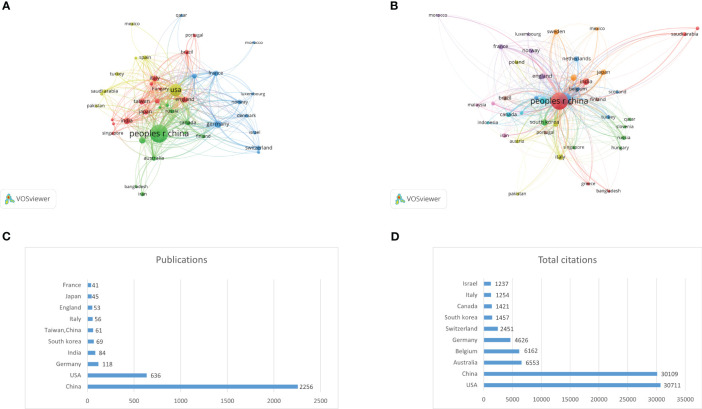
**(A)** Collaboration network of countries/regions. **(B)** Citation network of countries/regions. **(C)** Top 10 countries by publications. **(D)** Top 10 countries by total citations.

A total of 71 countries and regions have participated in research in this field. Among them, 43 countries have published at least 5 articles. We used VOSviewer software to analyse the cooperation and citation relationships (**Figures 3A, B**) and identify the top 10 countries with the highest number of publications (**Figure 3C**; **Table 1**) and citations (**Figure 3D**; **Table 2**). China (2256) has the highest number of publications, followed by the USA (635), Germany (118), India (84), South Korea (69), Taiwan, China (61), Italy (56), England (53), Japan (45), and France (41). The USA has the highest number of citations (30,711), followed by China (30,109), Australia (6553), Belgium (6162), Germany (4626), Switzerland (2451), South Korea (1457), Italy (1254), and Israel (1237).

**Table 1:** Top 10 most productive countries/regions.

Rank	Countries/Regions	Publications	Percentage	Total citations	Average citation per paper	H-index
**1**	China	2256	68.80146386	30109	13.3462	71
**2**	USA	636	19.39615737	30711	48.2877	71
**3**	Germany	118	3.598658127	4626	39.2034	31
**4**	India	84	2.561756633	1225	14.5833	19
**5**	South korea	69	2.104300091	1457	21.1159	20
**6**	Taiwan,China	61	1.860323269	913	14.9672	18
**7**	Italy	56	1.707837755	1254	22.3929	23
**8**	England	53	1.616346447	1159	21.8679	17
**9**	Japan	45	1.372369625	1186	26.3556	18
**10**	France	41	1.250381214	860	20.9756	20

**Table 2:** Top 10 most cited countries/regions.

Rank	Countries/Regions	Publications	Percentage	Total citations	Average citation per paper	H-index
**1**	USA	636	19.39615737	30711	48.2877	71
**2**	China	2256	68.80146386	30109	13.3462	71
**3**	Australia	32	0.975907289	6553	204.7812	14
**4**	Belgium	14	0.426959439	6162	440.1429	9
**5**	Germany	118	3.598658127	4626	39.2034	31
**6**	Switzerland	31	0.945410186	2451	79.0645	21
**7**	South korea	69	2.104300091	1457	21.1159	20
**8**	Canada	34	1.036901494	1421	41.7941	17
**9**	Italy	56	1.707837755	1254	22.3929	23
**10**	Israel	14	0.426959439	1237	88.3571	11

### Analysis of the distribution and cooperation of leading institutions

**Figure 4:**
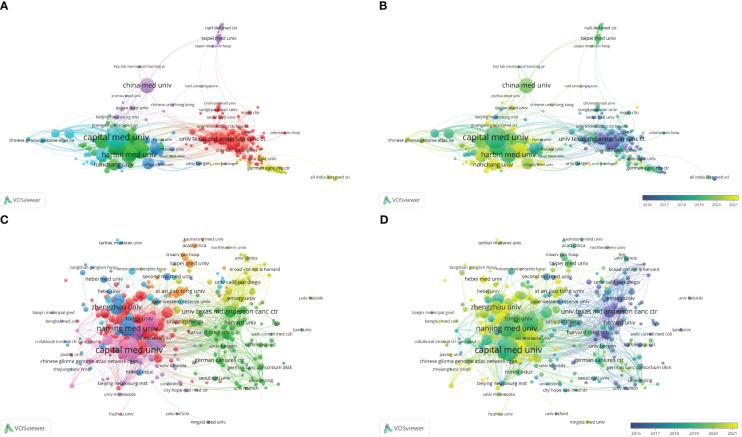
**(A)** Coauthorship analysis of institutions according to clusters. **(B)** The timestamp visualization of coauthorship among institutions. **(C)** Citation network of institutions according to clusters. **(D)** The timestamp visualization of relationships among institutions.

A total of 2805 institutions have participated in bioinformatics analyses of gliomas, of which 303 have published over 5 articles. We used VOSviewer software to analyse the cooperation relationships. Capital Medical University has the highest number of publications and citations, with an H-index of 36 (**Tables 3**, **4**). Nanjing Medical University is the second-largest institution in terms of publication volume, with an H-index of 30, followed by Harbin Medical University (H-index of 26). The top 10 institutions in terms of publication volume are all located in China (**Figures 4A, B**; **Table 3**). In the cluster analysis of cooperation among research institutions, those in Cluster1 were mainly in the USA, while those in Cluster2, Cluster3, and Cluster6 were mainly in China, those in Cluster4 were mainly in Germany, and those in Cluster5 were mainly distributed in the Chinese Mainland and Taiwan, China (**Figure 4A**; **Table S1**), indicating that scholars in various countries tend to collaborate domestically. Thus, international cooperation still needs to be strengthened. The citation relationships in terms of academic achievements between institutions also indicated a tendency for internal aggregation within the country, meaning that research institutions are more inclined to cite relevant literature from their own country (**Figure 4C**; **Table S2**). As shown in **Figures 4B, D**, most of the institutions with relatively recent average publication and citation dates are located in China, indicating that Chinese scholars have focused on bioinformatics analyses of gliomas in recent years.

**Table 3:** Top 10 most productive institutions.

Rank	Institutions	Publications	Total citations	Average citation per paper	H-index
**1**	Capital Medical University	213	4808	22.5728	36
**2**	Nanjing Medical University	124	2559	20.6371	30
**3**	Harbin Medical University	122	2635	21.5984	26
**4**	Central-South University	118	1012	8.5763	24
**5**	China Medical University	106	2322	21.9057	23
**6**	Fudan University	89	963	10.8202	17
**7**	Zhengzhou University	86	1012	11.7674	18
**8**	Southern Medical University	83	1053	12.6867	17
**9**	Shanghai Jiao Tong University	81	1167	14.4074	22
**10**	Sun Yat-sen University	78	1813	23.2436	22

**Table 4:** Top 10 most cited institutions.

Rank	Institutions	Publications	Total citations	Average citation per paper	H-index
**1**	Capital Medical University	213	4808	22.5728	36
**2**	The University of Texas MD Anderson Cancer Center	66	4600	69.697	35
**3**	Harvard University	27	4218	156.2222	34
**4**	Dana-Farber Cancer Institute	29	3917	135.069	22
**5**	Massachusetts Institute of Technology	8	3202	400.25	15
**6**	Harbin Medical University	122	2635	21.5984	26
**7**	Nanjing Medical University	124	2559	20.6371	30
**8**	Memorial Sloan-Kettering Cancer Center	24	2488	103.6667	21
**9**	University of North Carolina	11	2361	214.6364	10
**10**	China Medical University	106	2322	21.9057	23

### Analysis of coauthorships and citation relationships among authors

**Figure 5:**
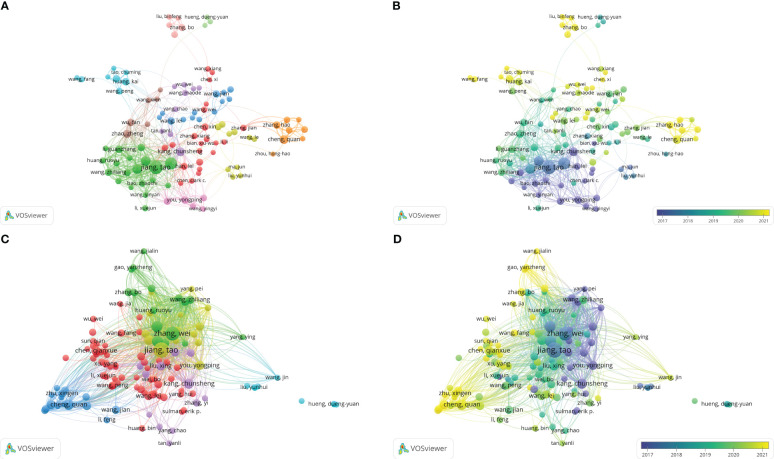
**(A)** Coauthorship analysis of authors according to clusters. **(B)** The timestamp visualization of coauthorship among authors. **(C)** Citation network of authors according to clusters. **(D)** The timestamp visualization of relationships among authors.

Between 2003 and 2022, 16,550 authors published literature on bioinformatics analysis on gliomas. According to Price’s law, the minimum publication volume of a core author is approximately 0.749 × N_max_^1/2^(N_max_ refers to the number of papers published by the author with the highest publication count during the statistical period), which was 6 in this study. We found 582 core authors in this field using VOSviewer software. Then, we plotted the collaboration and citation relationship networks of 122 authors with over 10 published articles (**Figure 5**). The 122 authors were divided into 11 clusters, with authors in each cluster cooperating more closely and could be considered a collaborative team (**Figure 5A**). As shown in **Figure 5B**, the clusters of Huang Kai and Wang Peng, Cheng Quan and Zhang Hao, and Liu Binfen and Liu Zhengdong published a large number of studies in recent years, indicating that these collaborative teams have focused substantially on bioinformatics analyses of gliomas in recent years.

The frequency of citations can reflect an author’s influence on the development of the field. **Figures 5C, D** show the network of citation relationships among authors. **Table 5** shows the top 10 authors with the highest number of publications and citations. Jiang Tao has the most published and cited articles. Wang Zheng, Zhang Chuanbao, Kang Chunsheng and You Yongping appeared in the top 10 productive and the top 10 cited author list, indicating that these scholars made substantial contributions to this field. Although Ma Jun and Zhang Junxia are not among the top 10 authors in terms of publication volume, the average number of citations per paper for them is significantly higher. In addition, You Yongping, Han Lei, Kang Chunsheng, and Zhang Chuanbao also have significantly higher average citation per paper (**Table 5**). The above results indicates that the articles written by Ma Jun, Zhang Junxia, You Yongping, Han Lei, Kang Chunsheng, and Zhang Chuanbao have relatively high quality.

**Table 5:** Top 10 most productive and cited authors.

Rank	Authors	Publications	Total citations	Average citation per paper	Rank	Authors	Publications	Total citations	Average citation per paper
**1**	Jiang, Tao	69	2725	39.4928	1	Jiang, Tao	69	2725	39.4928
**2**	Zhang, Wei	55	1931	35.1091	2	Zhang, Wei	55	1931	35.1091
**3**	Wang, Zheng	36	1445	40.1389	3	Zhang, Chuanbao	36	1619	44.9722
**4**	Zhang, Chuanbao	36	1619	44.9722	4	Wang, Zheng	36	1445	40.1389
**5**	Cheng, Quan	30	339	11.3	5	Kang, Chunsheng	29	1368	47.1724
**6**	Kang, Chunsheng	29	1368	47.1724	6	You, Yongping	23	1344	58.4348
**7**	Zhao, Zheng	25	817	32.68	7	Han, Lei	19	929	48.8947
**8**	You, Yongping	23	1344	58.4348	8	Zhang, Junxia	15	917	61.1333
**9**	Li, Guanzhang	22	842	38.2727	9	Ma, Jun	10	868	86.8
**10**	Liu, Xing	22	790	35.9091	10	Yan, Wei	20	846	42.3

### Analysis of cocited references

**Figure 6:**
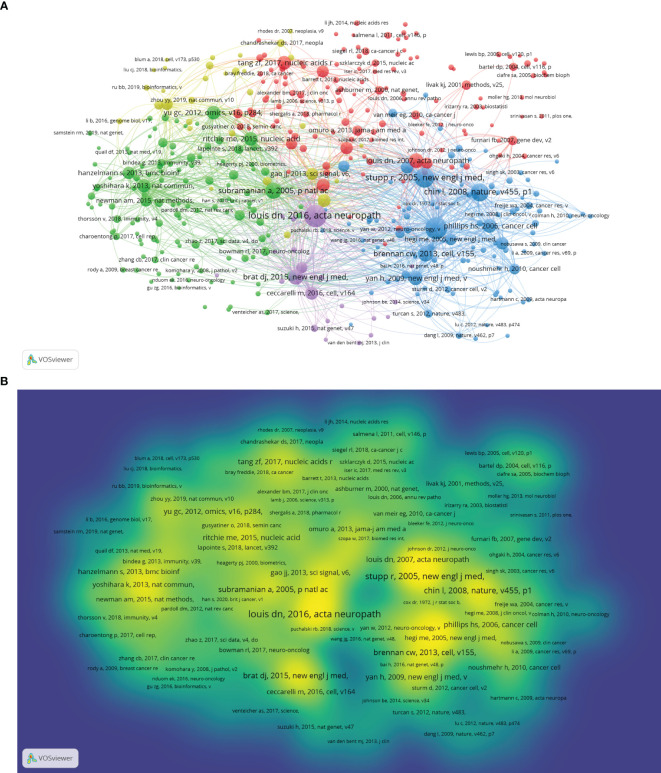
**(A)** Cocitation analysis of references according to clusters. **(B)** Density map of cocited analysis of references.

A total of 86,880 references were cited. We used VOSviewer software to visualize the cocitation network of 395 references cited over 20 times (**Figure 6A**). **Figure 6B** shows the intensity reference citations, with brighter colours indicating more frequent citations. **Table 6** lists the top 10 cited studies that form the foundation of glioma bioinformatics research. Among these 10 articles, 4 were from The Cancer Genome Atlas Research Network and involved the collection of glioma samples and analyses of core genes and pathways, indicating that The Cancer Genome Atlas (TCGA) database is crucial in glioma bioinformatics research. In addition, these 10 references mainly cover the subtypes and classification of glioma, molecular diagnostics, and bioinformatics data analysis methods, indicating that these factors are the foundation of glioma bioinformatics research. Roger Stupp et al. assessed the difference in prognosis between patients treated with radiotherapy combined with chemotherapy and those treated with radiotherapy alone, indicating that scholars in this field are interested in radiotherapy and chemotherapy outcomes in glioma patients.

**Table 6:** Top 10 most cited references.

Title	Authors	Publications	Country	Citations	TLS	Year
**The 2016 World Health Organization Classification of Tumors of the Central Nervous System: a summary**	David N. Louis et al	Acta Neuropathol	USA	568	4931	2016
**An integrated genomic analysis identifies clinically relevant subtypes of glioblastoma characterized by abnormalities in PDGFRA, IDH1, EGFR and NF1**	Roel G.W. Verhaak et al	Cancer Cell	USA	544	5043	2010
**Radiotherapy plus Concomitant and Adjuvant Temozolomide for Glioblastoma**	Roger Stupp et al	The new England journal of medicine	multiple countries	445	3534	2005
**Comprehensive genomic characterization defines human glioblastoma genes and core pathways**	The Cancer Genome Atlas (TCGA) Research Network	Nature	USA	374	3130	2008
**Gene set enrichment analysis: A knowledge-based approach for interpreting genome-wide expression profiles**	Aravind Subramaniana et al	PNAS	USA	302	2968	2005
**The Somatic Genomic Landscape of Glioblastoma**	TCGA Research Network	Cell	USA	278	2767	2013
**Comprehensive, Integrative Genomic Analysis of Diffuse Lower-Grade Gliomas**	The Cancer Genome Atlas Research Network	New England Journal of Medicine	USA	269	2839	2015
**The 2007 WHO ClassiWcation of Tumours of the Central Nervous System**	David N. Louis et al	Acta Neuropathol	USA	248	2059	2007
**IDH1 and IDH2 Mutations in Gliomas**	Hai Yan et al	New England Journal of Medicine	USA	235	2580	2009
**clusterProfiler: an R Package for Comparing Biological Themes Among Gene Clusters**	Guangchuang Yu et al	OMICS	China	219	2153	2012

### Burst references analysis

**Figure 7:**
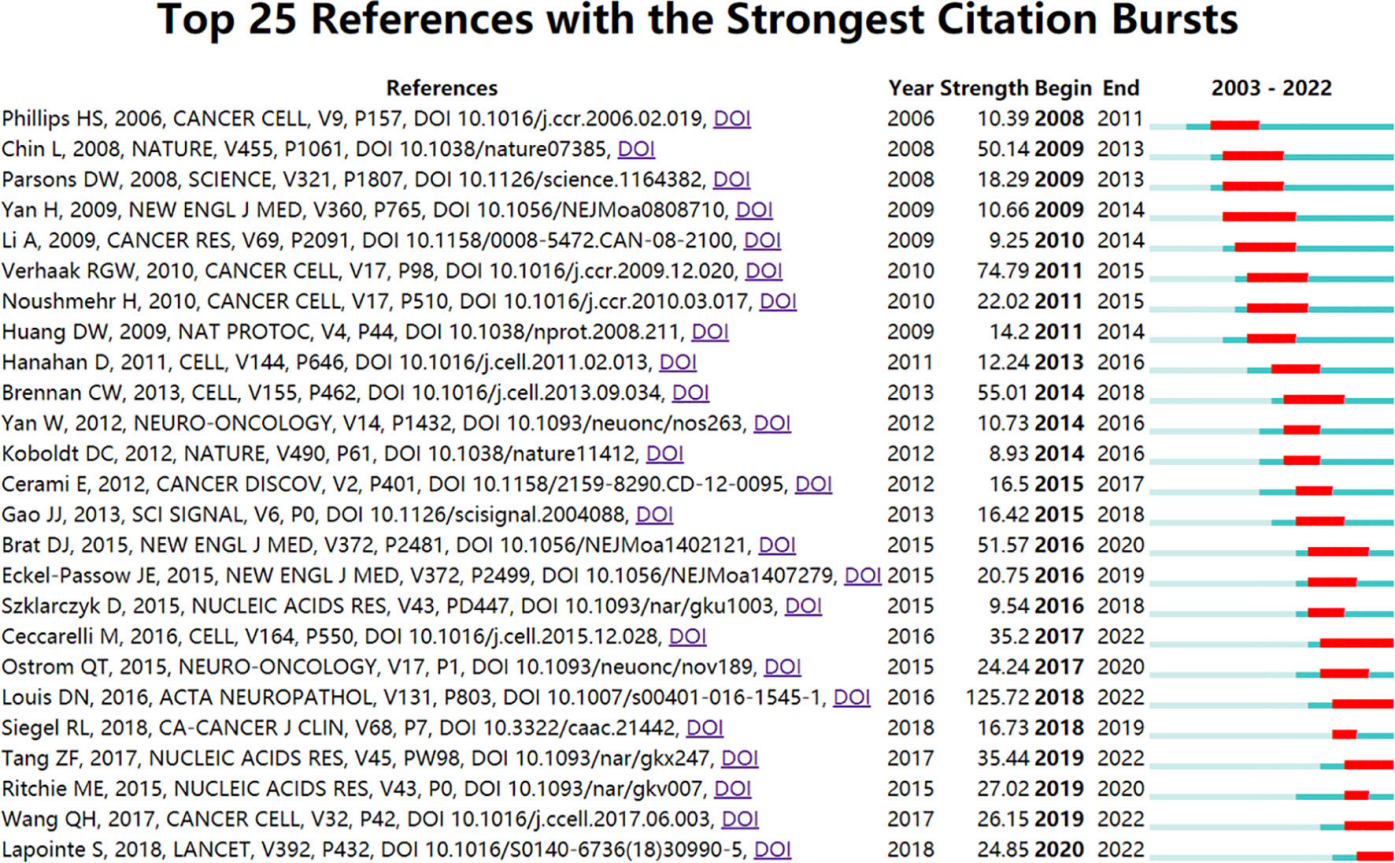
Visualization map of the top 25 references with the strongest citation bursts from 2003 to 2022.

The burst detection algorithm was invented by Kleinberg (41), and its calculation results can reflect the frontier and development trends of research over time. We used this algorithm to determine the key references related to glioma bioinformatics research. As shown in **Figure 7**, we used CiteSpace software to display the top 25 references with the strongest citation burst. Five references existed in the period of citation explosion in this field. These five studies mainly cover the following topics: the revised 2016 World Health Organization (WHO) classification of CNS tumours, which added new methods for molecular diagnosis; the immune microenvironment of glioma; further interpretation of glioma subtypes and discovery of new subtypes of gliomas, and the GEPIA (Gene Expression Profiling Interactive Analysis) database. This result indicates that scholars in this field tend to focus more on the molecular diagnosis and immune microenvironment of glioma and prefer to use the GEPIA database for analysis.

### Analysis of the development of the literature

**Figure 8:**
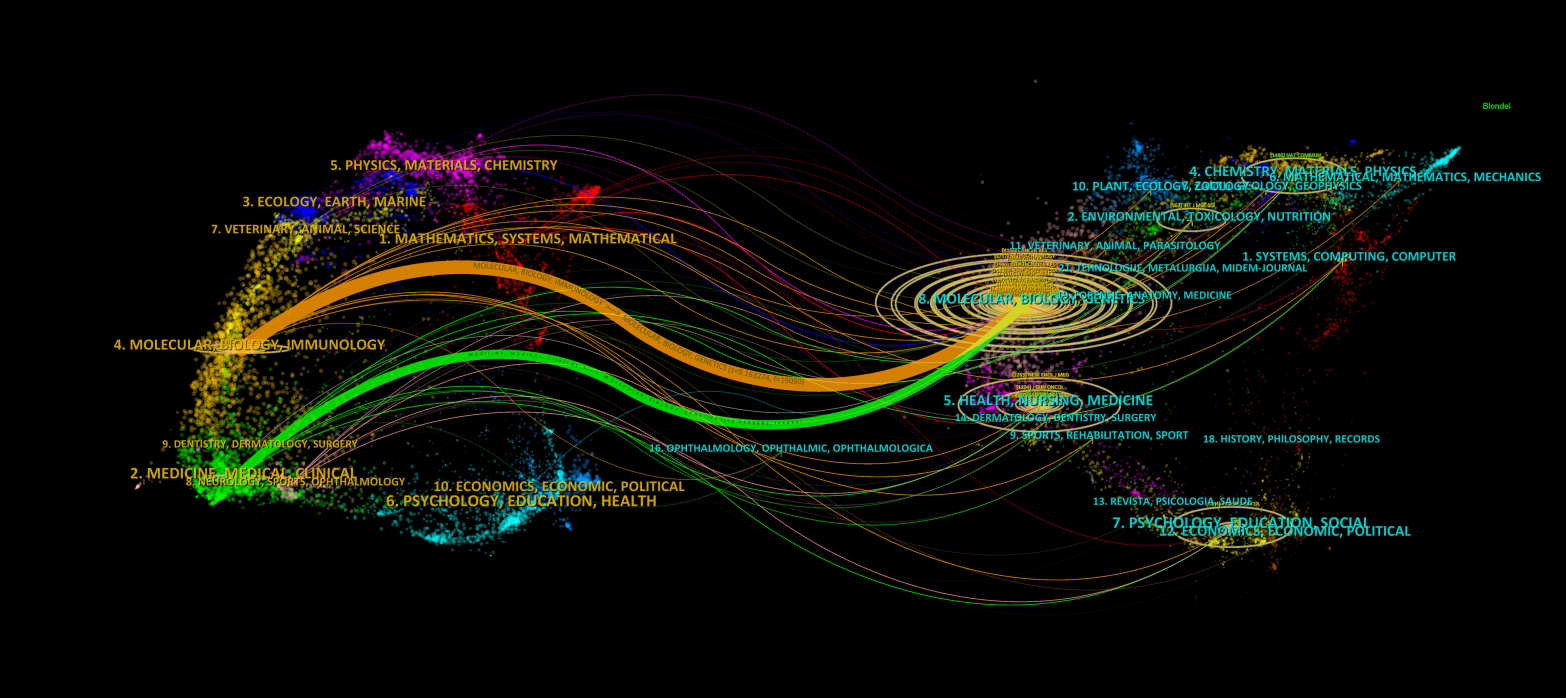
The dual-map overlay of journals. The more papers the journal publishes, the longer the vertical axis of the ellipse, and the greater the number of authors, the longer the horizontal axis of the ellipse.

Finally, we used the Dual-map Overlay function in CiteSpace to analyse the context of literature development in this field. As shown in **Figure 8**, we identified two main citation paths (orange and green). The orange path indicates that literature published in journals in the fields of molecular biology and genetics is often cited in journals in the fields of molecular biology and immunology, indicating that the study of tumour immune-related research is a relatively new research focus in glioma bioinformatics research, with high potential for exploration. The green path indicates that glioma bioinformatics research is moving from theory to application (i.e., from basic research to clinical practice) since studies published in journals in the fields of molecular, biology, and genetics are often cited in journals in the fields of medicine and medical and clinical research.

### Cluster analysis of co-occurring keywords

**Figure 9:**
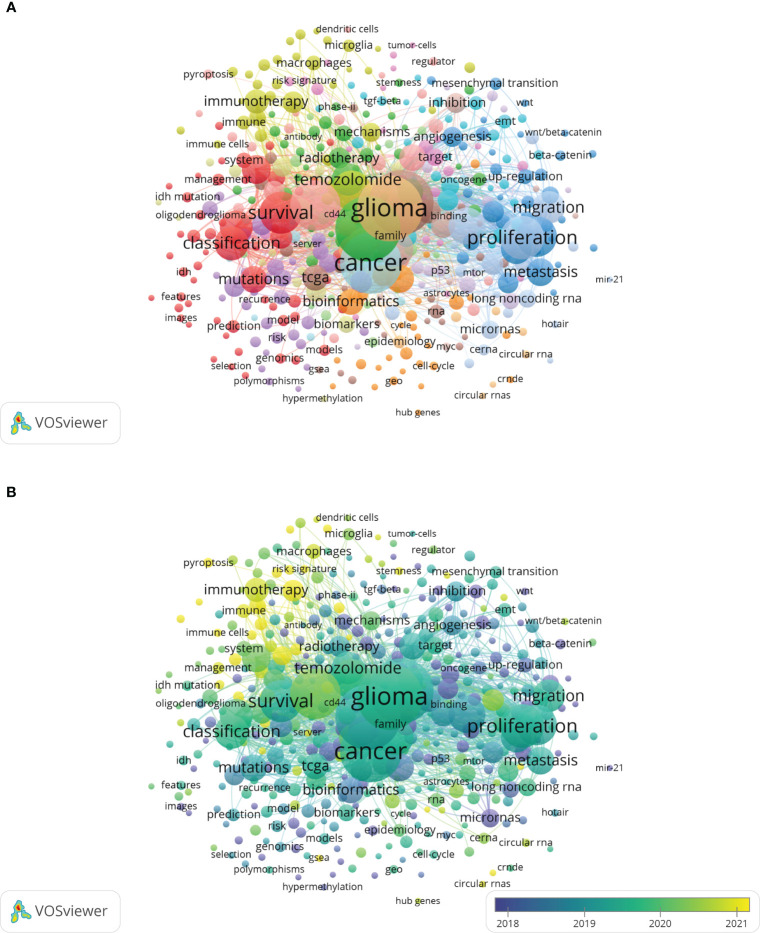
**(A)** Overlay map of keywords. **(B)** The timestamp visualization of keywords.

VOSviewer software was used to generate a keyword cooccurrence network (**Figure 9**). We set the minimum number of keyword occurrences to 10, and 435 keywords in the relevant literature met the criteria. The nodes and font sizes in **Figure 9A** represent the frequency of the keywords, reflecting their importance in the field. The keywords were divided into 17 clusters (**Figure 9A**; **Table S3**).

Cluster 1 includes keywords such as 1p/19q, IDH mutation, low-grade gliomas, astrocytoma, diagnosis, oligodendroglioma, prediction, prognostic marker, prognostic value, prognostic significance, and survival, indicating that this cluster may be related to the prognostic factors and clinically related indicators of glioma patients.

In cluster 2, keywords such as bevacizumab, chemoresistance, chemosensitivity, combination, drug resistance, MGMT promoter methylation, radiation therapy, radiosensitivity, radiotherapy, resistance, temozolomide, and trial indicate that the text in this section focuses on issues related to sensitivity, resistance mechanisms, and combination therapy in glioma regarding radiotherapy and chemotherapy and may involve clinical trials.

Cluster 3 keywords included adhesion, apoptosis, beta-catenin, cell invasion, cell proliferation, downregulation, *in vitro*, invasion, knockdown, upregulation and other commonly used terms in *in vivo* and *in vitro* research, suggesting that the texts represented by this cluster may be more focused on experimental verification after bioinformatics predictions.

Cluster 4 contains many immune-related keywords, such as immune, immune cells, immune checkpoint, immune microenvironment, immune response, immunity, immunosuppression, immunotherapy, macrophage, microenvironment, PD-1, and T cells, indicating that the text represented by this cluster is focused on the immune microenvironment of glioma and immunotherapy.

The appearance of words such as idh1, IDH2 mutations, subsets, subtypes, TP53, and EGFR in Cluster5 suggests that this cluster may involve research on glioma subtypes, classification, and genetic diagnosis.

Clusters 6-17 contained some commonly used terms related to bioinformatics data analysis, tumour genesis and development mechanism, classic tumour pathways, and noncoding RNA, indicating that the application of glioma bioinformatics analyses is extensive.

As shown in **Figure 9B**, the words appearing in recent years, such as tumour immune microenvironment, immune infiltration, immune cell infiltration, immunity, macrophage, immunosuppression, and PD-1, were all related to immunity, again indicating that the immune microenvironment of glioma has been the focus of scholars in recent years and is associated with good research prospects.

## Discussion

In this study, a bibliometric method was used to characterize and comprehensively analyse the 2003-2022 literature related to glioma bioinformatics, revealing the research status of this field and recent areas of focus. The number of publications in this field has steadily increased over the past 20 years, indicating the increasing application of bioinformatics methods in glioma research. The frequency of citations also indicated the rapid development of high-quality glioma bioinformatics analysis studies.

In terms of publishing countries, China had the largest total publication volume, and the USA had the largest total citation volume. However, from the perspective of the average number of citations per paper, Australia and Belgium demonstrated considerably higher numbers than China and the USA, indicating that the quality of the literature in these two countries was relatively better.

From the perspective of publishing institutions, the top 10 productive institutions were all in China, as were the majority of institutions with a relatively recent average publication year. Among the top 10 institutions with the highest number of citations, four were located in China, while the remaining 6 were in the USA. From the perspective of average citation per paper, it is evident that articles published by American institutions have significantly higher numbers of citations per paper (**Table 4**). These results indicated that research institutions in the USA have published high-quality literature in this field. Chinese scholars have focused extensively on this field in recent years; however, since Chinese scholars’ contributions are relatively recent, their publications have not received as much attention. The relatively short period in which Chinese scholars have focused on this field could also indicate a lack of experience, resulting in lower-quality literature. A lack of cross-border cooperation among institutions were found in our analysis. In fact, not only in the field of glioma research (42), but also in many other fields, bibliometrics studies have suggested researchers to break national boundaries and enhance communication and collaboration (43–45). This problem may be caused by language communication barriers and the long space distance. However, the exchange of ideas between countries is crucial for the development of human technology, and scholars should enhance their awareness of cross-border communication. In addition, Capital Medical University had the highest number of publications and citations, representing significant contributions to glioma bioinformatics research.

Regarding the authors, we statistically analysed 582 core authors focused on glioma bioinformatics-related research, reflecting the significant interest of scholars in this field. Jiang Tao, Wang Zheng, Zhang Chuanbao, Kang Chunsheng, and You Yongping have significantly impacted this field, appearing in the top 10 productive and top 10 cited author lists. Ma Jun, Zhang Junxia, You Yongping, Han Lei, Kang Chunsheng, and Zhang Chuanbao had a higher article quality with a higher average citation per paper.

In the analysis of references, we found that the TCGA database has significantly contributed to glioma bioinformatics research. Scholars in this field require knowledge of glioma classification, molecular typing, current treatment measures, and bioinformatics data analysis methods. In addition, research on glioma immunity has been a research focus in this field in recent years, and the GEPIA database is a popular analytical tool among scholars. The citation path analysis revealed that glioma bioinformatics analysis research is moving from theory to application and from basic research to clinical practice.

The keyword analysis of literature related to glioma bioinformatics analysis from 2003 to 2022 revealed that the current application of bioinformatics in gliomas mainly focuses on using bioinformatics methods to perform differential analyses, conduct network analyses on omics data, and establish clinical prognosis models. This field covers a wide range of topics, including the analysis of the sensitivity of glioma cells to radiotherapy and chemotherapy, diagnosis of glioma at the genetic level, immunotherapy, subtype analysis, stemness research, phenotype transformation analysis, pathway analysis, and experimental validation. In the keyword analysis, we again found that the current research focus is on tumour immune microenvironment research and immunotherapy. In the past, the concept of a tumor was merely a simple aggregation of abnormally proliferating cells. Now, it has been realized that tumors are composed of various components known as the tumor microenvironment, which forms a highly organized ‘organ’ (46). The immune components in the tumor microenvironment are referred to as the tumor immune microenvironment (TIME), including innate immune cells, adaptive immune cells, extracellular immune factors, and cell surface molecules (47–49). They have unique internal interactions and play an important role in tumor biology. With the development of single-cell sequencing technology in bioinformatics, by extracting, reverse transcription, amplifying, and sequencing at the single-cell level, we can obtain the gene characteristics of a specific cell type and better reveal the composition of various immune components in the tumor microenvironment (50).

Based on the comprehensive analysis above, the author has made the following recommendations on the research directions and considerations for collaboration in this field for reference by researchers (1). Collecting more clinical samples and enriching the content of the database are the foundation for further development of bioinformatics analysis (2). It is suggested to enhance the interpretation of tumor immune microenvironment and analyze the mechanisms of immunotherapy (3). It is recommended to enhance the in-depth interpretation of glioma subtypes (4). In cooperation, it is important to pay attention to enhancing collaboration and communication between teams, especially multinational teams. It is advisable to refer to research achievements from other countries and broaden research ideas.

Although we have conducted a relatively comprehensive analysis of glioma bioinformatics research from 2003 to 2022, there are inevitably some limitations (1). When screening the literature, we limited the language and time frame, resulting in some non-English articles and new articles published in 2023 not being included, which may lead to potential bias (2); Our literature was obtained from the WOSCC core database, which may have overlooked some relevant literature in other databases (3); VOSviewer and CiteSpace software do not provide all advanced statistical functions, which to some extent limits personalized analysis (4); Bibliometric analysis has the advantage of analyzing a wide range of time and space, but it is often difficult to control the quality of the literature analyzed (5).In literature screening, considering that review articles also play an important role in advancing the discipline, we have included such literature in our analysis. But review articles will only include articles published before itself, which may cause some bias. However, the purpose of this study is to conduct high-quality bibliometric analysis on the bioinformatics research of glioma, and these limitations do not affect the current research results.

## Conclusion

Bioinformatics research on gliomas showed a trend of increasing popularity between 2003 and 2022. Many countries and institutions are participating in this research; however, international cooperation among institutions is relatively lacking in comparison with domestic cooperation. Research in this field is broad and covers almost all basic research fields associated with glioma; additionally, this field is moving towards clinical applications, such as drug development and targeted personalized treatment. The current focus in the field of glioma bioinformatics is immune-related research.

## Data availability statement

The original contributions presented in the study are included in the article/**Supplementary Material**. Further inquiries can be directed to the corresponding authors.

## Author contributions

Conceptualization, XL and PZ. Methodology, XY and MD. Software, XY and MD. Formal analysis, XY and MD. Writing—original draft preparation, XY. Writing—review and editing, XL and PZ. Visualization, XY and MD. Supervision, XL and PZ. Project administration, XL and PZ. Funding acquisition, XL. All authors contributed to the article and approved the submitted version.

## Supplementary material

Supplementary Table 1 Clusters of coauthorship among organizations.

Supplementary Table 2 Clusters of citation relationships among organizations.

Supplementary Table 3 Clusters of keywords.
